# Mechanism of Chromium Separation and Concentration from Tannery Wastewater by Membrane Methods

**DOI:** 10.3390/membranes13030295

**Published:** 2023-02-28

**Authors:** Paweł Religa, Bernadetta Kaźmierczak

**Affiliations:** 1 Faculty of Chemical Engineering and Commodity Science, Kazimierz Pulaski University of Technology and Humanities in Radom, Chrobrego 27, 26-600 Radom, Poland; 2 Bioeconomy and Ecoinnovation Centre, Proecological Technologies Research Group, Łukasiewicz Research Network, Institute for Sustainable Technologies, Pulaskiego 6/10, 26-600 Radom, Poland

**Keywords:** nanofiltration, diafiltration method, tannery wastewater, chromium, circular economy

## Abstract

The article specifies conditions for a nanofiltration process employing a diafiltration method with a constant volume diafiltration (NF-CVD) for exhausted chromium tannery wastewater treatment and describes a mechanism of the examined process carried out for model wastewater. The authors prove that a decrease in salt concentration in the NF-CVD process is an important factor that enables effective concentration of chromium. Based on the proposed chromium separation and concentration mechanisms, it was found that this effect may be achieved by (1) limiting the formation of an ionic adsorption–polarization layer and (2) reducing the increase in the osmotic pressure caused by a change in the separation properties of the membrane. The article shows that in the analyzed system a higher amount of the solvent introduced at the diafiltration stage and a lower process pressure that ensures a reduction in salt retention translate to a high level of salt removal. In regenerates, after the NF-CVD processes in which at least the same volume of a washing diluent as the volume of the retentate after the pre-concentration step was used, salt concentration below 10 g L^−1^ and chromium concentration about three times higher than in the case of the feed solution were obtained. Therefore, the proposed solution implements the circular economy strategy in the tannery.

## 1. Introduction

In the tannery industry, apart from the desired product, which is leather, a large amount of wastewater is also generated. The most troublesome wastewater from tanneries are exhausted chromium tannery baths characterized by low pH (above 4) and high concentrations of chromium(III) and chloride salts. The amount of this hazardous wastewater ranges from 15 to 80 m^3^ per tonne of rawhides [1,2,3,4]. The chemical precipitation method is commonly used for the disposal of chromium tannery wastewater [5,6,7,8,9,10]. This method involves introducing appropriate substances to the wastewater which causes precipitation of chromium(III) hydroxide. Unfortunately, chromium sludge from the chemical precipitation process contains, apart from chromium, many other substances present in tannery wastewater, primarily organic compounds. This composition of the sludge, due to the possibility of causing stains on the leather, disqualifies it from being reused in the tanning process and means that it becomes hazardous waste. In addition, the obtained effluent contains a high concentration of salts, which reduces the efficiency of its further biological purification process. Another method for neutralizing pre-treated chromium tannery wastewater may be the use of pressure membrane processes, particularly nanofiltration [11,12,13,14,15]. The importance of this process in the case of chromium tannery wastewater, which is a mixture of monovalent and polyvalent ions with different concentrations, results from the properties of nanofiltration membranes having functional groups on the surface and in the internal structure. These groups, so-called active centers, are endowed with an electric charge. In this case, the mechanism of separation of chromium components of tanning wastewater in the NF process includes not only the “sieve effect”, but also takes into account the “electrostatic effect” [16,17]. The charged membrane creates ideal conditions for the transport of oppositely charged monovalent ions. This effect is additionally enhanced by the presence of large polyvalent ions in the chromium tanning wastewater. With the increase in the concentration of these ions, caused by their high retention and the system’s desire to maintain electroneutrality, the degree of retention of monovalent ions decreases. This phenomenon is commonly known as the Donnan effect. Therefore, the use of NF allows partial separation of salt from the inexhaustible chromium in the leather tanning process. The final result was a permeate containing a high salt concentration and practically without chromium. The final retentate was characterized by the presence of chromium and, unfortunately, a high concentration of salts. The obtained permeate can be reused in the process, at the stage of hide pickling. On the other hand, the use of a retentate as a regenerated tanning bath, due to the presence of salt concentrations above 10 g∙L^−1^ and a relatively low chromium content, is difficult for technological reasons [18]. The recycled retentate as a regenerated tanning bath suitable for reuse in the tanning process should have a salt concentration below 10 g∙L^−1^ and a possibly high chromium concentration. Therefore, it would be advantageous to conduct the nanofiltration process in such a way as to remove salt from the retentate to a greater extent. The literature reports [19,20,21,22] prove that carrying out the nanofiltration process by means of diafiltration increases the effect of separating salts from macromolecular compounds present in the solutions subjected to filtration. Diafiltration is a method for conducting filtration which involves the introduction of a solvent (usually water) to the pre-concentrated retentate in order to wash out low-molecular-weight compounds. The degree of salt washing varies from 75%, when the volume of the added solvent is twice as high as the volume of the retentate after the pre-concentration step [19], up to 95–99% when this volume is multiplied [20,21,22].

In the case of pre-treated chromium tannery wastewater, the final separation in the nanofiltration process mainly involves ionic components of various sizes—polyvalent chromium and sulfate ions, and monovalent chloride and sodium ions. Therefore, it was assumed that the combination of nanofiltration and diafiltration processes would ensure their effective separation. The aim of the work was, therefore, to investigate the mechanisms of separation and concentration of chromium in exhausted tanning baths in the process of nanofiltration employing the diafiltration method, and to determine the process conditions that will enable the production of a regenerate—a concentrated chromium solution with a reduced salt content, which will allow its reuse. This concept of the process of regeneration of exhausted chromium tanning baths is fully consistent with the concept of circular economy [23,24,25]. The introduction of the proposed solution to the operation of tanneries may contribute to reducing their negative impact on the environment, by saving both water and raw materials.

## 2. Materials and Methods

The tests were carried out in a laboratory installation described in [26] with a flat-sheet membrane module containing a flat type nanofiltration membrane by GE Osmonics, made of polyamide, with an active surface of 0.0155 m^2^ and permeability coefficient of 1.5 × 10^−6^ m^3^/m^−2^∙s^−1^∙bar^−1^. According to the manufacturer’s data [27], the limit molar mass of the membrane is in the range of 150–300 g·mol^−1^. Therefore, it constitutes a theoretically impermeable barrier for polyvalent ions, while monovalent ions and water freely permeate through it. The membrane used was also characterized by an isoelectric point (IP) value of 3. In the tested solution (pH 4), the membrane had a negative initial charge with a potential of about −14 mV [28].

The processes were carried out for model exhausted chromium tanning baths with the following composition: 10 g SO_4_^2−^/L^−1^, 20 g Cl^−^/L^−1^, and 2 g Cr^3+^/L^−1^. The low pH of the model solution creates conditions in which chromium occurs in the Cr^3+^ speciation. This chromium speciation is required for the leather tanning process.

The model baths were prepared from anhydrous sodium sulfate, sodium chloride, and chromium(III) chloride hexahydrate. The selected salts were dissolved in deionized water. Each time, the volume of the model exhausted chromium tanning baths was 4 L. The temperature was kept constant at 20 °C in the tested systems during the process.

In order to achieve the assumed research objective, the nanofiltration process (NF) and the nanofiltration process by diafiltration were carried out with a constant solvent dosing rate (NF-CVD) equal to the rate of the received permeate. The NF-CVD process consisted of three steps: (1) pre-concentration; (2) diafiltration, during which assumed volumes of solvent were introduced to the retentate obtained after the pre-concentration step; and (3) final concentration, involving final concentration of the retentate. The pre-concentration step was carried out until the permeate flux decreased by about 20% compared to the initial value. This level of pre-concentration provided satisfactory efficiency of the diafiltration step. The diafiltration step was carried out in three variants differing in the total amount of diluent introduced. The permeate flux remained constant at this stage. In the case of both the nanofiltration by diafiltration method and nanofiltration, the final concentration stage was completed with a decrease in the permeate flux by about 60% compared to its initial value. Based on the discussion of the previous results [28,29], it was shown that further concentration of the model exhausted chromium tanning bath adversely affects the separation properties of the membrane and may lead to irreversible (partial) loss of its permeability.

The processes were carried out in cross-flow with recirculation of the feed. Such a way of conducting the processes resulted in an increase in the concentration of ions in the feed, which favored the polarization of the membrane. In order to reduce the impact of this unfavorable phenomenon during the process, the retentate flow was maintained at a level of 800 [L·h^−1^], which ensured the turbulent nature of its flow. In addition, a spacer (0.8 mm) was also used, which is an additional factor increasing turbulence in the system. The driving force of the processes was the pressure of 10 bar. The value of the applied pressure was only slightly higher than the osmotic pressure of the solution, which was estimated at 9 bar based on the van ’t Hoff equation. The assumed value of pressure ensured both satisfactory efficiency of the process and a high degree of salt permeability through the membrane.

After each process, the membrane was initially rinsed with tap water and then washed with deionized water. In order to regenerate the membrane, it was first soaked in an HCl solution with pH = 3, and then, after rinsing with tap water, in an NaOH solution with pH = 11. After the regeneration process, the membrane was rinsed with tap water and then with deionized water. The membrane was stored in a vessel with deionized water. Such a method of membrane washing, regeneration, and storage enabled its repeated use without loss of initial permeability.

Mohr’s titration method was used to determine the concentration of chloride ions present in the solution. Spectrophotometric analysis with 1,5-diphenylcabazide was used to determine the concentration of chromium(III) ions. The pH and conductivity of the obtained baths were also controlled.

Based on the performed determinations and measurements, the following were determined:

The chromium retention:

(1) $$R=\left(1-\frac{C_{p}}{C_{N}}\right)\;\cdot 100\%\;$$

where:

R—chromium retention, [%],
C_N_—chromium concentration in the feed, [g∙L^−1^],
C_P_—chromium concentration in the permeate, [g∙L^−1^].

Degree of salt washing:

(2) $$E=\left(1-\frac{C_{\mathrm{SR}}}{C_{\mathrm{SN}}}\right)\cdot 100\%$$

E—degree of salt washing, %,
C_SR_—salt concentration in the retentate after the process, [g∙L^−1^],
C_SN_—salt concentration in the feed, [g∙L^−1^].

The feed reduction factor:

(3) $$\mathrm{VRF}=\frac{V_{N}}{V_{R}}$$

where:

V_N_—feed volume, [L],
V_R_—volume of retentate after the process, [L].

The chromium concentration factor:

(4) $$\mathrm{CF}=\frac{C_{R}}{C_{N}}$$

where:

C_R_—chromium concentration in the retentate after the process, [g∙L^−1^].

## 3. Results

### 3.1. Nanofiltration

**Figure 1:**
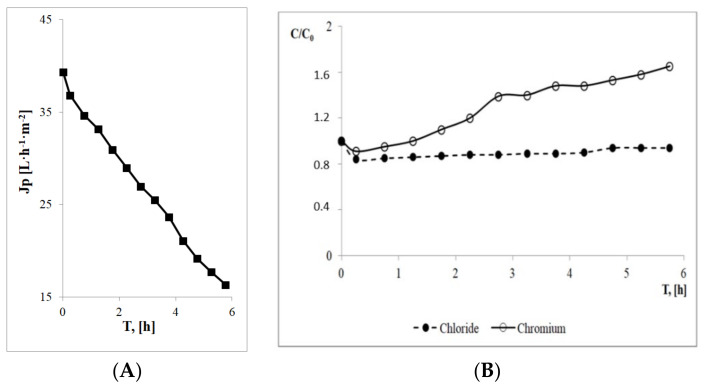
Changes in: (**A**) the permeate flux and (**B**) ion concentrations in the retentate over time for the NF process.

As part of the preliminary research, the nanofiltration process was carried out for model exhausted chromium tanning baths. After 6 hours of the process, the permeate flux decreased by 60%, compared to the initial value (Figure 1A). In accordance with the adopted assumptions, the process was interrupted. The final retentate stream obtained during this process contained chromium ions and significant amounts of chloride ions (Figure 1B). The permeate obtained was a salt solution with a negligibly low concentration of chromium from the functional perspective.

**Figure 2:**
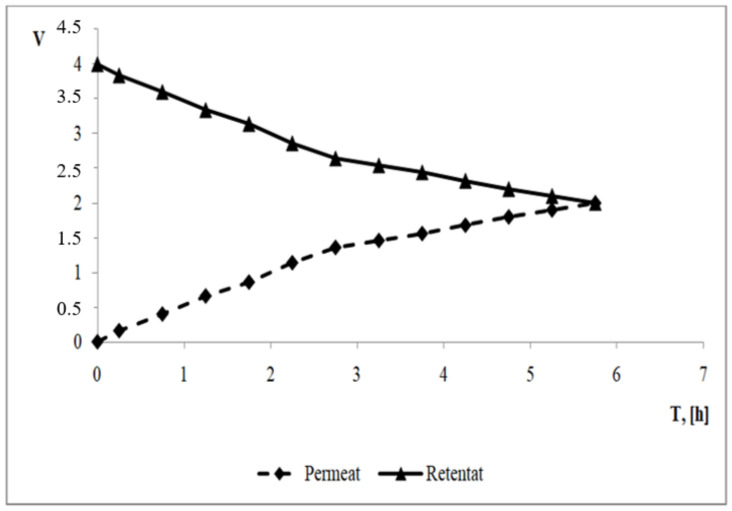
Water balance for the NF process.

A water balance was also prepared (Figure 2). For the nanofiltration process, the final retentate and permeate volumes were the same. The total final volume of water was equal to the volume of the feed.

Under the assumed conditions, the classic nanofiltration of model chromium tannery wastewater was to reduce the wastewater volume only by half and to obtain the final concentration of chromium(III) at the level of 3.30 g∙L^−1^ and the salt concentration of 18.30 g∙L^−1^ (Table 1). Unfortunately, the composition of the obtained retentate, characterized by a low chromium concentration and a high salt content, does not show the characteristics of a technologically useful bath. In addition, its significant volume increases the cost of its possible disposal. Such a composition of the final retentate is a consequence of a small level of feed volume reduction and a small degree of salt washing of only 9% (Table 1).

### 3.2. Nanofiltration by Diafiltration (NF-CVD)

The aim of the next stage of the research was to determine the effect that changing the method of conducting the nanofiltration process would have on the concentration of chromium in the retentate. Model exhausted chromium tanning baths were nanofiltered by diafiltration at a constant solvent dosing rate (NF-CVD). Based on previous research results [29], it was assumed that for the nanofiltration process carried out by CVD diafiltration, only a low (about 20%) initial concentration of the model chromium solution would be beneficial, ensuring high efficiency of the diafiltration stage. The process was carried out for three variants, which differed in the amount of solvent introduced at the diafiltration stage (Table 2).

**Figure 3:**
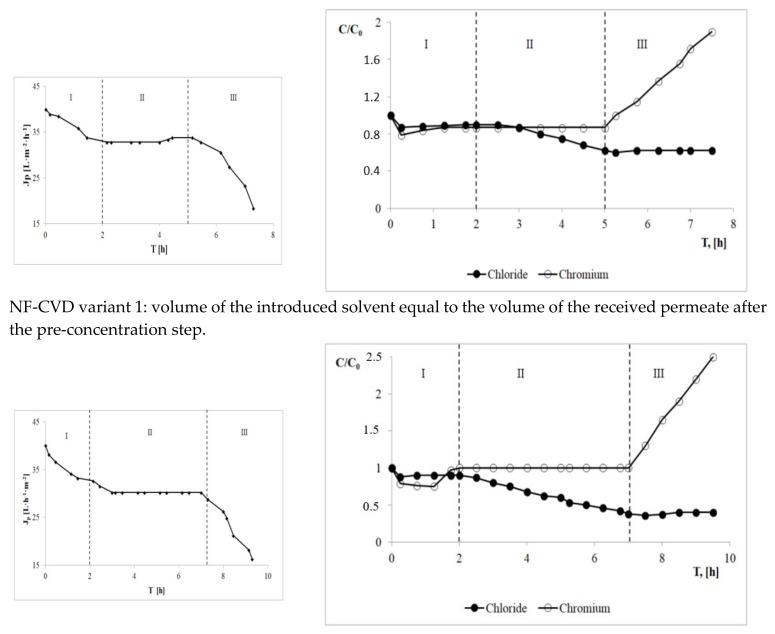

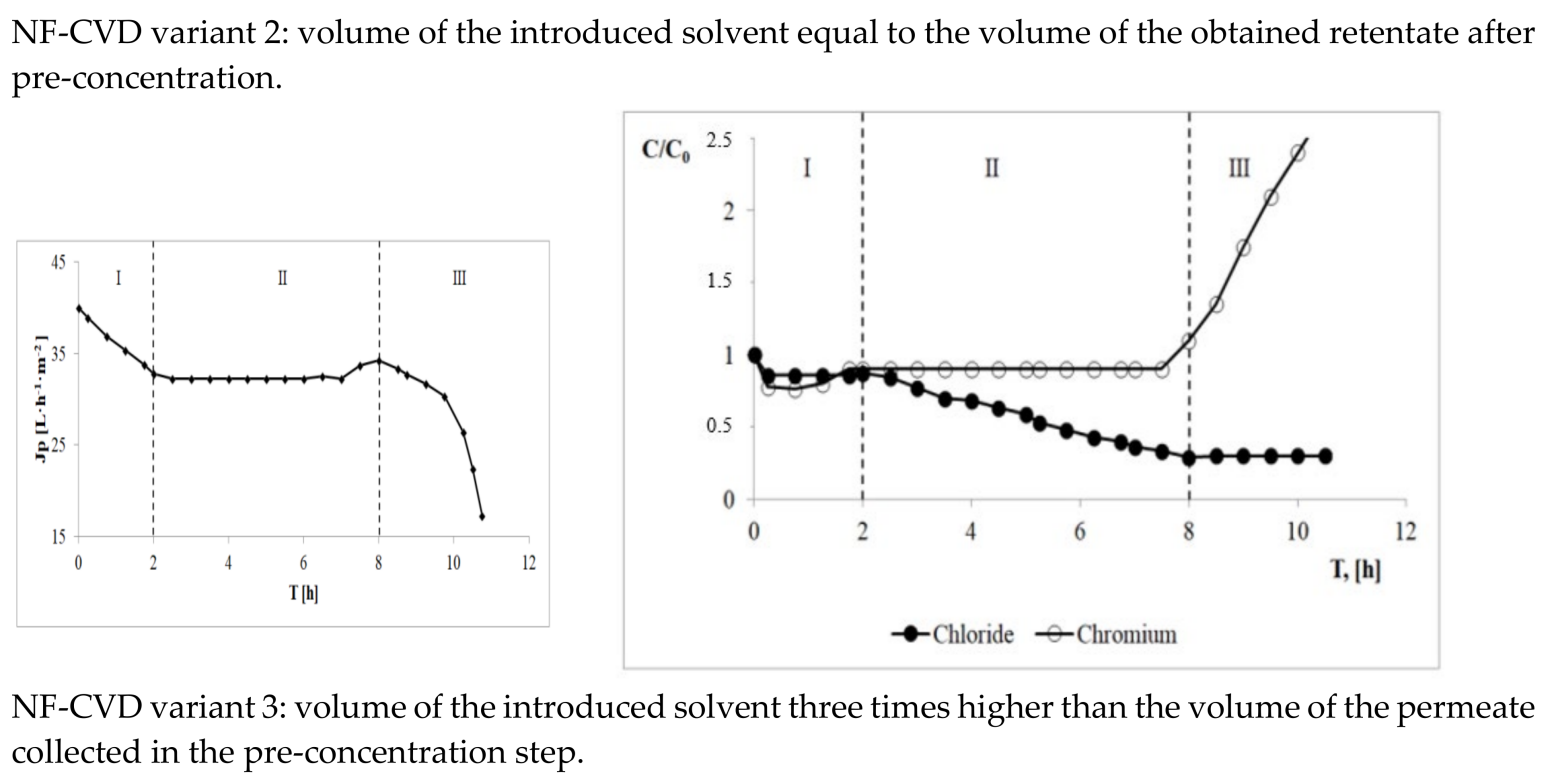
Changes in permeation flux and ion concentrations in the permeate and retentate over time for NF-CVD processes carried out with pre-concentration. I—Pre-concentration stage; II—Diafiltration stage; III—Final concentration stage.

During the NF-CVD process, changes in the permeation flux and concentration of chromium(III) ions and chloride ions over time were observed in the retentate for each variant (Figure 3). In the case of the same small reduction in the permeation flux and the volume of the feed in the initial concentration stage, the concentration of chromium in the retentate slightly decreased, for each variant, and then systematically, although slowly, increased. However, the concentration of chloride ions did not change. In the diafiltration stage, solvent was added in an amount equal to the amount of the permeate received. This way of conducting the process allowed a constant permeate flux to be maintained. At this stage, in each process variant, the concentration of chromium(III) ions in the retentate remained at a constant level. On the other hand, the concentration of chloride ions in the retentate was gradually decreasing. In this step, the final salt concentration in the retentate was lower the more solvent was added. A different relationship was observed in the final concentration step. During the final concentration, the concentration of chromium in the retentate increased. At the same time, the increase in the concentration of chromium in the retentate was greater, the greater the degree of salt washing out at the diafiltration stage. Changes in the permeate flux were also observed at this stage. When no more solvent was added, the permeate flux value decreased, particularly for lower levels of salt washing in the retentate, in which case it decreased at a quicker rate. For each variant, the final concentration stage was completed with a decrease in the permeate flux by about 60%, compared to its initial value. In the case of salt, no significant changes in its concentration in the retentate were observed at the final concentration stage.

The composition of the final retentates obtained was characterized by a much higher amount of chromium than the initial amount and a lower amount of salt, depending on the amount of water added in the diafiltration stage (Table 2). However, in the variant of the NF-CVD process 1 where the volume of the introduced solvent was equal to the volume of the received permeate after the pre-concentration step, the salt concentration decreased only from 20.0 g∙L^−1^ to about 12.5 g∙L^−1^. The concentration stage in this variant lasted a relatively short time due to the rapid decrease in the efficiency of the process, which resulted in a relatively large final retentate volume of 1.6 L. The small reduction in the feed volume caused the chromium concentration in the final retentate to be 3.81 g∙L^−1^. For variant 2 of the NF-CVD process, where the volume of the introduced solvent was equal to the volume of the obtained retentate after pre-concentration, the final salt concentration in the retentate was 7.65 g∙L^−1^. The significant reduction in the salt concentration in the diafiltration stage enabled the volume of the feed to be reduced almost three times. As a result, the final concentration of chromium in the retentate was 4.93 g∙L^−1^. In variant 3 of the NF-CVD process, at the diafiltration stage, the solvent was introduced to the pre-concentrated retentate in a volume over three times larger than the volume of the permeate collected in the pre-concentration step. The consequence of such an action was a final concentration of chlorides in the retentate at the level of 5.81 g∙L^−1^. As a result, during the final concentration step, it was possible to reduce the volume of the retentate more than three times, and obtain a final concentration of chromium in the retentate of 5.48 g∙L^−1^.

As in the case of chromium concentration in the nanofiltration process, in this case, in all variants, the permeates obtained were more or less dilute salt solutions with a negligibly low chromium concentration from the functional perspective.

**Figure 4:**
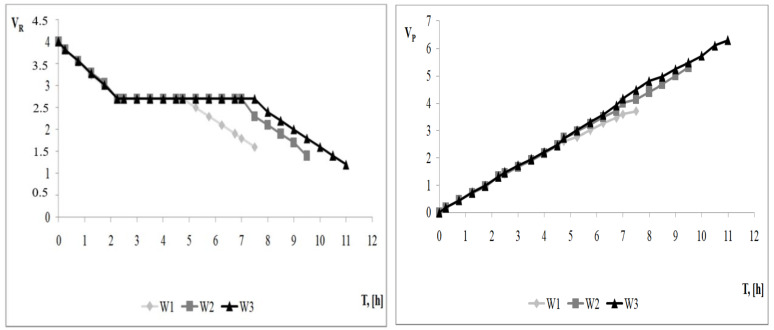
Water balance of retentate and permeate for NF-CVD processes. W1—NF-CVD 1; W2—NF-CVD 2; W3—NF-CVD 3.

Water balances were obtained for the processes carried out (Figure 4). These balances differ in the total final volumes of permeate and retentate, depending on the variant of the NF-CVD process. These differences result from the volume of solvent introduced in the diafiltration step. In NF-CVD process 3, the highest total volume of retentate and permeate was obtained. In this variant, the largest difference between the final volume of permeate and retentate was also observed. The higher volume of the obtained permeate for this variant of the process results from the introduction of higher volumes of the solvent to the system at the diafiltration stage. The introduction of higher volumes of the solvent also created favorable conditions for reducing the volume of the retentate. The smallest total volume of retentate and permeate streams was obtained for NF-CVD 1. In this variant, the lowest final feed volume reduction and the smallest difference between the final volume of permeate and retentate were also observed.

The nanofiltration process carried out by the CVD diafiltration method enabled both a more favorable decrease in the salt concentration and an increase in the concentration of chromium in the retentate, compared to the nanofiltration process. The composition of the obtained retentates in the variants of the NF-CVD process, in which at least the same solvent volume as the retentate volume after pre-concentration was used, meets the requirements of a regenerated tanning bath. Salt concentration below 10 g∙L^−1^ was obtained in these baths. The concentration of chromium was also increased about three times. The retentates—as regenerated tanning baths—can thus be reused in the process. Such a composition of the final retentate is a consequence of a high level of feed volume reduction, a high chromium retention at the level of 93-95%, and a high degree of salt washing at the level of 62–71% (Table 2).

## 4. Discussion

**Figure 5:**
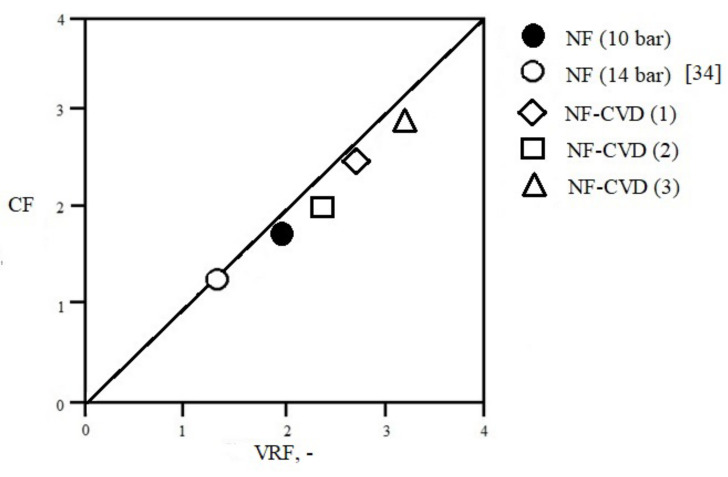
Correlation between the volume reduction factor (VRF) and the chromium(III) concentration factor (CF) for NF and NF-CVD processes.

The obtained test results indicate that the higher the level of salt removal at the diafiltration stage of regeneration of model chromium tanning wastewater through nanofiltration, the more favorable the degree of reduction of the feed volume and final effect of chromium concentration (Figure 5). Therefore, the presence of high concentrations of salt in the tested solution not only has a significant adverse effect on the possibility of obtaining baths of a specific composition, but negatively impacts on the efficiency of the nanofiltration process [30,31,32,33,34].

**Figure 6:**
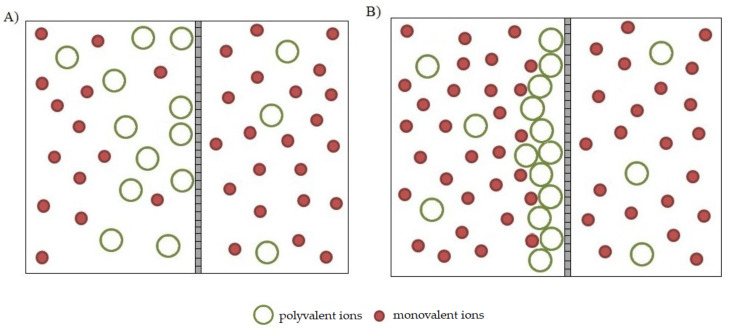
Mechanism of separation of monovalent and polyvalent ions in the nanofiltration process of model chromium tannery wastewater. (**A**) Process initiation and pre-concentration stage; (**B**) final concentration stage.

In the nanofiltration process, the membrane is a separation barrier for polyvalent ions, but it is permeable for monovalent ions (Table 1). Therefore, at the initial stage of the chromium concentration process in the model tannery wastewater, monovalent ions permeate freely through the membrane, while multivalent ions, including chromium ions, are retained (Figure 6A). The presence of highly charged multivalent ions close to the surface of nanofiltration membranes, characterized by the presence of active ionic groups, leads to the interaction of these ions with the membrane. In such systems, as indicated in the works of Bruni et al. [35] and Bandini et al. [36], favorable conditions for the adsorption of ions present in the solution are created on the membrane surface. The adsorbed ions create a barrier that hinders the flow of the solvent as well as the permeation of monovalent ions. The confirmation of the described phenomenon is also the result of the work of Religa et al. [28] on the application of the nanofiltration process for the separation of chromium from saline solutions. In addition, with the increasing degree of concentration of polyvalent ions, they form a polarizing layer at the membrane surface, increasing the resistance of the membrane and changing its separation properties (Figure 6B). The consequence of this, especially in the case of solutions with a high concentration of monovalent ions, may be co-precipitation of these ions with polyvalent ions and scaling of the membrane. Moreover, an increase in salt retention can cause an increase in osmotic pressure in the system and an additional decrease in process efficiency. One more unfavorable consequence of the high concentration of polyvalent ions, including chromium ions, near the membrane is their facilitated transport through it, which contributes to a decrease in their retention. In the case of nanofiltration, the chromium retention is only 83% (Table 1).

**Figure 7:**
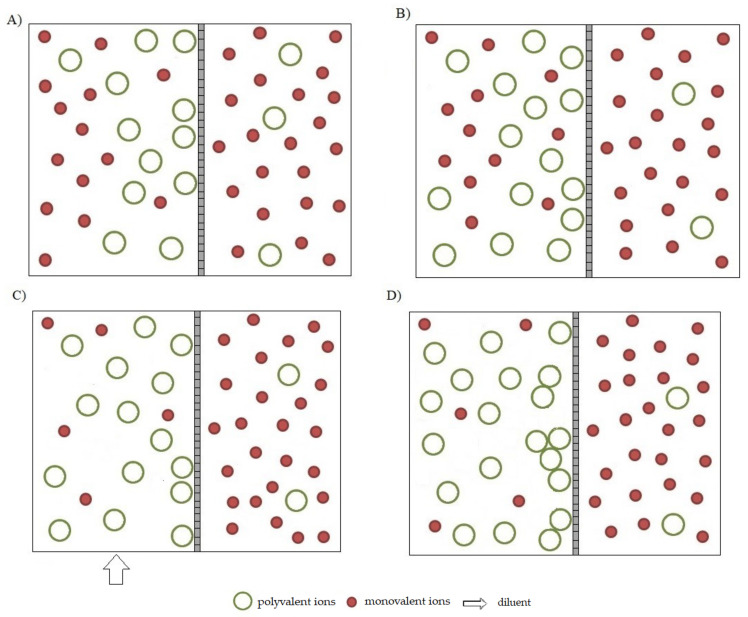
Mechanism of separation of monovalent and polyvalent ions in the nanofiltration process by diafiltration of model chrome tannery wastewater. (**A**) Process initiation stage; (**B**) Pre-concentration stage; (**C**) diafiltration stage; (**D**) final concentration stage.

Conducting the nanofiltration process using the diafiltration method, involving the introduction of a solvent to the pre-concentrated feed in order to wash out low-molecular-weight substances, limits the problems of an additional decrease in the efficiency of the process caused by scaling and an increase in osmotic pressure during chromium concentration in model tannery wastewater. Similarly to the nanofiltration process, at the initial stage of the NF-CVD process, monovalent ions permeate freely through the membrane, while polyvalent ions, including chromium ions, are retained (Figure 7A). Additionally, the presence of highly charged polyvalent ions close to the surface of nanofiltration membranes, characterized by the presence of active ionic groups, similarly leads to the interaction of these ions with the membrane’s ionic groups and formation of an adsorption layer that hinders the flow of the solvent as well as the permeation of monovalent ions. In this case, however, the pre-concentration stage is carried out only until a slight increase in the concentration of polyvalent ions (Figure 7B) preceding the formation of the polarizing layer in the system occurs. This method of conducting the process also has a positive effect on chromium retention, which in this case is at the level of 93–95% (Table 2). At the next stage—diafiltration—a solvent is added, which causes the washing of salts from the system (Figure 7C). The level of salt washout can be adjusted by the amount of the solvent added (Table 2). Removal of the salt from the system eliminates the participation of the salt in the polarization of the membrane and formation of an ionic adsorption layer on its surface. It is also of critical importance to the elimination of the osmotic pressure in the system, which was the case in the nanofiltration process. Therefore, favorable conditions are created for more effective concentration of polyvalent ions at the final concentration stage (Figure 7D).

Therefore, lowering the salt concentration in the NF-CVD process is a factor that allows for a much more effective separation and concentration of chromium in model tannery wastewater. This effect can be achieved by limiting the formation of the ionic adsorption–polarization layer and limiting the increase in osmotic pressure caused by the change in the separation properties of the membrane. A high level of salt leaching, as indicated by the test results (Figure 3) and the results of other works [26,37], is favored by a higher amount of solvent introduced at the diafiltration stage. In the research context, therefore, the highest degree of salt removal is advantageous, because with the increase in the degree of solution desalting, an increase in the degree of chromium concentration is observed. However, it should be noted that salt washing involves the introduction of solvent and the formation of larger amounts of permeate (Figure 4) salt solution (permeate also contains washing water added at the diafiltration stage), which is a by-product in the absence of a reuse process. Hence, in the context of the practical use of the obtained results, the fact is that in the retentate formed in the NF-CVD process of the exhausted chromium tanning baths, the salt concentration can be reduced to any level, and thus also to a technologically acceptable level. Given the above, the proposed solution enables recirculation of the regenerated tanning bath. The factor limiting the application of the proposed solution in real conditions, after meeting the requirement to reduce the salt concentration in the retentate to a technologically acceptable level, will therefore be the amount of the permeate that can be reused in the tannery. However, it should be remembered that obtaining a high level of chromium concentration in the regenerate obtained in the NF-CVD process will be favored by a higher level of salt washing. Such an approach should be considered in the case of utilization of regenerate by the precipitation method. In this case, the efficiency of the process is higher the higher the concentration of chromium in the retentate. 

The study also compared the final characteristics of the tested system for the nanofiltration process carried out at two different values of transmembrane pressure, i.e., 14 bar and 10 bar (Figure 5). The results of the research indicate that the factor increasing the level of chromium concentration in the model tannery wastewater is a lower value of the transmembrane pressure. Probably, the reason for such results is the lower level of salt retention, and thus a higher degree of salt removal in the system when a lower transmembrane pressure was used. This supposition is confirmed by the results of other works [15,28,38,39], clearly indicating that the retention of monovalent ions increases with increasing pressure. In the case of lower pressure, due to the lower permeate flux, the mechanism of diffusion transport of ions through the membrane plays a dominant role. At high salt concentrations, this mechanism is extremely effective. Therefore, an additional reduction of the salt concentration in the tannery wastewater in the nanofiltration process is favored, by the reduction of the process pressure, which ensures a lower level of salt retention.

## 5. Conclusions

The research carried out aimed to provide the necessary information to propose a solution that would allow for the effective separation of the main components of tannery wastewater—salt and chromium—and their reuse in the process. To date, the results of work on the use of the nanofiltration process for this purpose allow only a partial solution to the problem, i.e., the reuse of permeate, which is a salt solution, in the pickling process. The obtained retentate, characterized by a low concentration of chromium and a high content of salt, unfortunately does not show satisfactory features of a technologically useful bath. In addition, its significant volume increases the cost of its possible disposal.

As part of this work, research was carried out on the nanofiltration process and nanofiltration using the CVD diafiltration method for model chromium tannery wastewater. The results of the research showed that the presence of high concentrations of salts in spent chrome tanning baths is an important factor that not only has a negative impact on the possibility of obtaining baths with a specific composition, but also reduces the efficiency of the nanofiltration process. The reason for the identified adverse effect of salt is its retention. Salt retention in the tested systems is caused by the adsorption–polarization layer, which is formed by polyvalent ions on and near the membrane. This barrier significantly hinders salt permeation. The consequence of this is the co-precipitation of these ions with polyvalent ions and scaling of the membrane. In addition, an increase in salt retention can cause an increase in the osmotic pressure in the system and an additional decrease in process efficiency. Carrying out the nanofiltration process using the diafiltration method in the case of regeneration of exhausted chromium tanning baths enables the washing out of chloride ions, while maintaining high chromium(III) retention, which allows for its effective concentration.

The proposed solution allows for (1) more than a three-fold reduction in the volume of the exhausted chromium tanning bath; (2) obtaining an almost three times higher concentration of chromium in the obtained regenerate. In addition, from a practical point of view, with the use of the NF-CVD process for the regeneration of chromium tannery wastewater, a level of salt washing from the regenerate below 10 g∙L^−1^ can be obtained. This level of salt in the regenerate is acceptable from a technological point of view. The study shows that lowering the salt concentration at the diafiltration stage below the required value is possible after adding to the regenerated bath an amount of solvent at least equal to the volume of the retentate obtained after the pre-concentration stage. Therefore, the proposed solution enables the recirculation of exhausted chromium tanning baths, which in turn makes it more possible to close the water and raw material cycles (chromium and salt) at the leather tanning stage.

## Figures and Tables

**Table 1:** Characteristics of nanofiltration process parameters.

Parameter	Nanofiltration
Feed volume, L	4.0
Final retentate volume, L	2.0
Final permeate volume, L	2.0
Initial concentration of chlorides, g∙L^−1^	20
Final concentration of chlorides in the permeate, g∙L^−1^	16.87
Final concentration of chlorides in the retentate, g∙L^−1^	18.30
Degree of salt washing, %	9
Initial concentration of chromium, g∙L^−1^	2
Final concentration of chromium in the permeate, g∙L^−1^	0.33
Final concentration of chromium in the retentate, g∙L^−1^	3.30
Chromium retention, %	83
Process time, h	5.75

**Table 2:** Characteristics of the NF-CVD process carried out for a constant pre–concentration level and various amounts of washing diluent added in the diafiltration step.

Parameter	NF-CVD		
	NF-CVD (1)	NF-CVD (2)	NF-CVD (3)
Feed volume, L	4.0	4.0	4.0
Retentate volume after pre-concentration stage, L	2.7	2.7	2.7
Final retentate volume, L	1.6	1.4	1.2
Final permeate volume, L	3.7	5.3	6.3
Solvent volume (deionized water), L	1.3	2.7	3.5
Initial concentration of chlorides, g∙L^−1^	20		
Final concentration of chlorides in the retentate, g∙L^−1^	12.48	7.65	5.81
Final concentration of chlorides in the permeate, g∙L^−1^	14.46	12.76	11.42
Degree of salt washing, %	38	62	71
Initial concentration of chromium, g∙L^−1^	2		
Final concentration of chromium in the retentate, g∙L^−1^	3.81	4.93	5.48
Final concentration of chromium in the permeate, g∙L^−1^	0.10	0.14	0.14
Chromium retention, %	95	93	93
Process time, h	7.25	9.75	11
